# Sustainable Anisaldehyde-Based Natural Deep Eutectic Solvent Dispersive Liquid–Liquid Microextraction for Monitoring Antibiotic Residues in Commercial Milk and Eggs: A Comprehensive Evaluation of Greenness, Practicality, Analytical Performance and Innovation

**DOI:** 10.3390/foods15020258

**Published:** 2026-01-10

**Authors:** Heba Shaaban, Ahmed Mostafa, Abdulmalik M. Alqarni, Marwah Alsalman, Makarem A. Alkhalaf, Mohammad A. Alrofaidi, Abdulaziz H. Al Khzem, Mansour S. Alturki

**Affiliations:** 1 Department of Pharmaceutical Chemistry, College of Pharmacy, Imam Abdulrahman Bin Faisal University, King Faisal Road, Dammam 31441, Saudi Arabia; ammostafa@iau.edu.sa (A.M.); amalqarni@iau.edu.sa (A.M.A.); ahalkhzem@iau.edu.sa (A.H.A.K.); msalturki@iau.edu.sa (M.S.A.); 2 College of Pharmacy, Imam Abdulrahman Bin Faisal University, Dammam 31441, Saudi Arabia; mostafaabdelrahman05@gmail.com (M.A.); ziad.mostafa.5403@gmail.com (M.A.A.); 3 Department of Pharmaceutical Chemistry, Faculty of Pharmacy, Al-Baha University, Al-Baha P.O. Box 1988, Saudi Arabia; malrofaidi@bu.edu.sa

**Keywords:** natural deep eutectic solvents, NADES, sustainable chemistry, dispersive liquid-liquid microextraction, DLLME, green analytical chemistry, GAC, greenness assessment, antibiotics, LC-MS/MS

## Abstract

The widespread use of antibiotics in human medicine, veterinary care, and livestock production has resulted in their frequent detection in diverse environmental and food matrices, making continuous surveillance of antibiotic residues in food products essential for consumer protection. In this study, a sustainable analytical method based on dispersive liquid–liquid microextraction (DLLME) coupled with UHPLC–MS/MS was developed for the trace determination of sulfamethoxazole, sulfadimethoxine, and enrofloxacin in commercial cow milk and chicken eggs. A natural deep eutectic solvent (NADES) composed of anisaldehyde and octanoic acid (2:1, molar ratio) was employed as a biodegradable extraction solvent, and key extraction parameters were systematically optimized. Under optimized conditions, the method demonstrated excellent linearity (R^2^ ≥ 0.9982), recoveries of 89.5–98.7%, and RSDs ≤ 6.04%. Application to 44 commercial samples from the Saudi market revealed sulfamethoxazole as the most frequently detected antibiotic, occurring in 90% of egg samples (2.17–13.76 µg kg^−1^) and 70.8% of milk samples (0.26–26.67 µg L^−1^). A comprehensive evaluation using ten metrics confirmed the method’s greenness, practicality, analytical performance, and innovation. Overall, the proposed NADES–DLLME–UHPLC–MS/MS approach offers a rapid, cost-effective, and environmentally friendly alternative for routine monitoring of antibiotic residues in food matrices.

## 1. Introduction

In recent decades, increasing attention has been given to making pharmaceutical analysis more environmentally sustainable through the careful design of analytical methods that minimize adverse effects on both human health and the environment [1]. Sample preparation is a critical stage in numerous analytical methods, designed to eliminate matrix interferences, concentrate target analytes, or facilitate derivatization. Conventional pharmaceutical analytical techniques are often regarded as environmentally detrimental because they rely on large volumes of hazardous organic solvents, leading to significant chemical waste generation [2,3,4,5]. One of the key principles of green analytical chemistry (GAC) is the miniaturization of analytical procedures to reduce organic solvents consumption [6]. Dispersive liquid–liquid microextraction (DLLME) is a downsized sample preparation approach that has been widely applied across various analytical chemistry fields [7]. This technique offers several advantages over conventional extraction methods, including operational simplicity, cost-effectiveness, ease of use, and rapid processing. Nevertheless, the careful choice of dispersing and extracting solvents remains a significant challenge [8]. Traditional DLLME approaches often rely on hazardous solvents which pose risks to human health and the environment [9]. Adopting safer solvent alternatives aligns with the principles of GAC should be a priority during method development. Consequently, environmentally friendly deep eutectic solvents (DESs) have recently emerged as a sustainable option for DLLME applications [10]. In general, DESs consist of two or more compounds, typically hydrogen bond donors and acceptors, combined in a specific ratio to form a homogeneous mixture with an eutectic point below that of its constituents [11]. With the aim of making the analytical process more sustainable, DLLME utilizing natural DES is considered an effective approach for fulfilling the twelve principles of GAC. These solvents are relatively non-toxic, biodegradable, accessible, and simple to prepare. Recently, NADES have been widely used in various fields, including food, environmental and cosmetics analysis, e.g., [12,13,14,15]. Anisaldehyde-based NADES have previously been developed and applied by our research group for the selective extraction of different analytes such as parabens and bisphenols [13]. Unlike highly polar choline chloride- or betaine-based DES, which preferentially extract strongly polar compounds, p-anisaldehyde provides an aromatic and moderately hydrophobic environment. Its aromatic ring and aldehyde functionality enable π–π interactions and hydrogen bonding with less polar analytes, enhancing extraction selectivity and improving phase separation. Antibiotic resistance has become a pressing global health threat, responsible for an estimated 0.7 million deaths annually, a number projected to reach 10 million by 2050 [16]. The widespread use of antibiotics in human medicine, veterinary care, and livestock production has led to their frequent detection in diverse environmental and food matrices. These compounds have been reported in surface water, groundwater, wastewater, and agricultural soils, mainly through manure application and irrigation with contaminated water, facilitating their entry into the food chain [17]. Contamination has also been observed in food products such as milk, meat, eggs, and fish, particularly due to their use in animal husbandry and aquaculture. The administration of antibiotics to food-producing animals results in drug residues accumulating in animal-derived products [18]. Such residues promote the development of antimicrobial-resistant bacteria in animals, which may spread to the environment and eventually reach humans. Beyond therapeutic purposes, antibiotics are frequently administered prophylactically to prevent infections or, in some cases, as growth promoters [19]. Poor farm management practices, lack of hygiene, and indiscriminate use of these drugs further exacerbate residue levels. Residues can persist in edible tissues, milk, eggs, and skin during the withdrawal period, which varies with the type of antibiotic used [20]. Prolonged exposure to low concentrations of antibiotic residues in humans has been linked to allergic reactions, disruption of gut microbiota, and even increased cancer risk [21]. Maintaining the safety and integrity of foods of animal origin is crucial for safeguarding public health. Protecting consumers requires ongoing surveillance of antibiotic residues in food products, which is of utmost significance.

The present work aims to develop and optimize a green NADES-DLLME-UHPLC-MS/MS method for the determination of sulfamethoxazole and sulfadimethoxine (sulfonamides) and enrofloxacin (a fluoroquinolone) in 20 chicken eggs and 24 cow milk samples purchased from the Saudi market. The selected antibiotics were chosen as target analytes due to their extensive use in poultry and dairy production, their limited metabolic degradation, and their frequent detection as residues in milk and eggs. Compared with other antibiotic classes, these compounds show higher persistence and mobility in animal-derived foods and are prioritized in residue monitoring programs due to their strong association with antimicrobial resistance and food safety risks.

To verify the compliance of the proposed method with greenness standards, its greenness profile was assessed. The utilization of multiple assessment tools can provide a holistic overview of the eco-friendless level of the proposed method [22]. In this context, six assessment tools were used: Analytical Eco-Scale (AES) [23], Green Analytical Procedure Index (GAPI) [24], Analytical GREEnness Metric (AGREE) [25], Analytical Greenness Metric for Sample Preparation (AGREEprep) [26], Modified GAPI (MoGAPI) [27], and the newly introduced Analytical Green Star Area (AGSA) [28]. Additionally, the applicability and practicality of the developed method was assessed using the Blue Assessment of Green Index (BAGI) [29] and the Click Analytical Chemistry Index (CACI) [30]. Furthermore, the analytical performance and innovation level of the developed method was evaluated using Red Analytical Performance Index (RAPI) [31] and Violet Innovation Grade Index (VIGI) [32], respectively.

## 2. Materials and Methods

### 2.1. Materials

Standards of sulfamethoxazole, sulfadimethoxine and enrofloxacin (purity ≥ 99%) were obtained from Sigma-Aldrich (Steinheim, Germany). Ethanol of analytical grade was supplied by Merck (Darmstadt, Germany). Acetic acid, formic acid, octanoic acid, oleic acid, thymol, menthol were purchased from Sigma-Aldrich (Steinheim, Germany). p-Anisaldehyde (4-methoxybenzaldehyde, ≥99%) from Sigma-Aldrich (Steinheim, Germany) was used as the hydrogen bond donor in the NADES formulation. Stock solutions of the target analytes (1000 mg L^−1^) were prepared in ethanol and stored at 4 °C. Working solutions were freshly prepared by diluting the stock solutions and kept at −20 °C until use. A stock solution of the isotopically labeled internal standards, sulfamethoxazole (Phenyl-^13^C_6_) and ofloxacin-d3 purchased from Sigma-Aldrich (Steinheim, Germany), were prepared by dissolving 5 mg in 5 mL of ethanol and stored at −20 °C. Ultrapure water employed throughout the study was produced using a Pure Lab Ultra system (ELGA, High Wycombe, UK).

### 2.2. Instrumentation

Separation of analytes was carried out using a Shimadzu Nexera X2 UHPLC system (Shimadzu, Kyoto, Japan) interfaced with an 8050 triple quadrupole mass spectrometer (Shimadzu, Kyoto, Japan) equipped with an electrospray ionization (ESI) source and operated using LabSolution^@^ Insight version 3.5 from Shimadzu (Kyoto, Japan). The analysis was carried out on an Acquity BEH C18 column (2.1 mm × 100 mm, 1.7 µm; Waters, Milford, MA, USA) fitted with a guard pre-column (Waters, Milford, MA, USA) and maintained at 40 °C. The flow rate was 0.3 mL min^−1^. The injection volume was 2 µL. The gradient elution started at 15% solvent B, increased linearly to 30% at 1.50 min and to 90% at 4.50 min, followed by a 2-min equilibration. Prior to use, the mobile phase was degassed by sonication for 15 min in a Branson 5510 ultrasonic bath (Danbury, CT, USA).

For mass spectrometric analysis, multiple reaction monitoring (MRM) was conducted in positive ion mode. MRM transitions for each analyte were optimized through flow injection analysis and the automated MRM optimization feature in LabSolutions^®^ software (Version 3.5). After selecting the precursor ions for each analyte, product ion scans were carried out to determine the most abundant fragment ions. Identification of the analytes was based on their LC retention times and the ratio of two MRM transitions, with a tolerance of ±20% relative to the reference standards. Once a target analyte was positively identified, quantification was performed using the most intense MRM transition. The detailed optimized MRM parameters are provided in Table 1.

**Figure 1:**
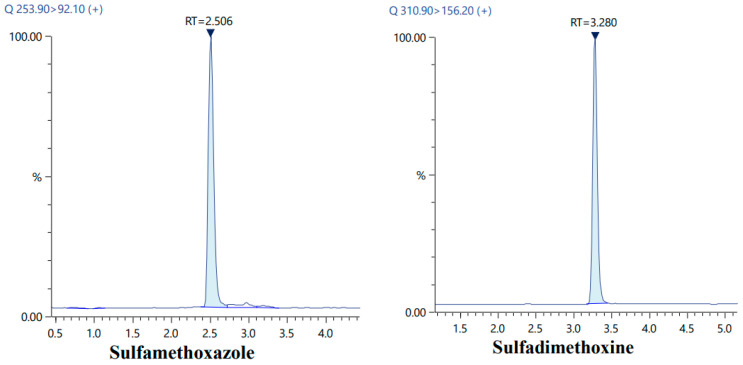

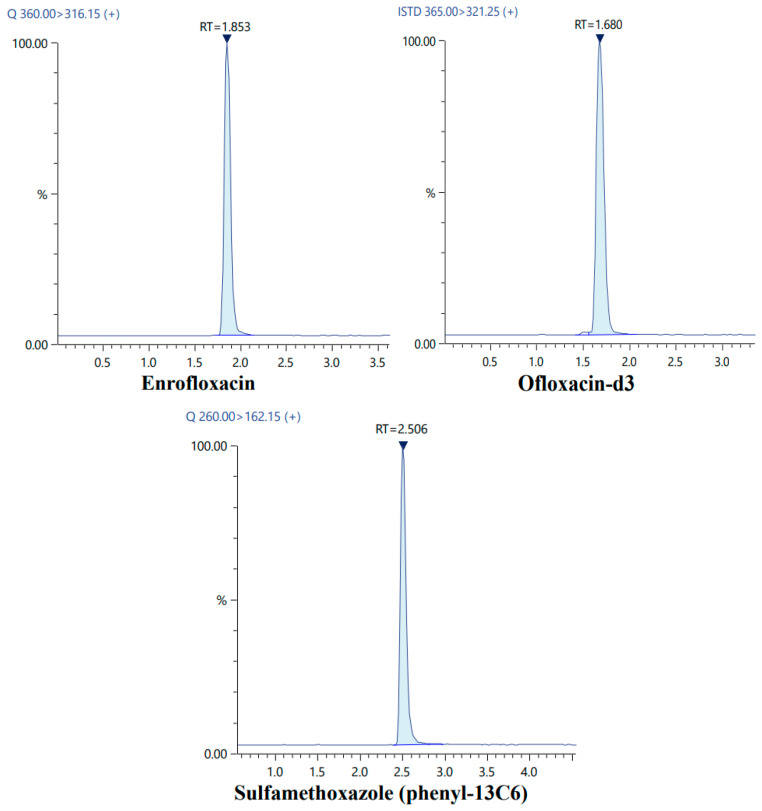
LC-MS/MS transition chromatograms of sulfamethoxazole, sulfadimethoxine, enrofloxacin, sulfamethoxazole (Phenyl-^13^C_6_) and ofloxacin-d3.

The electrospray ionization (ESI) source was operated at 300 °C, with the desolvation line and heat block maintained at 250 °C and 400 °C, respectively. Air was supplied as the heating gas at a flow rate of 10 L min^−1^, whereas nitrogen was used as both the nebulizing gas (3 L min^−1^) and the drying gas (10 L min^−1^). Argon gas (Airgas, Radnor, PA, USA) was employed for collision-induced dissociation in the collision cell. Target analytes were identified based on their LC retention times and the ratio of two MRM transitions, compared against reference standards with a 20% tolerance. Upon positive identification, quantification was carried out using the most intense MRM transition. A representative UHPLC-MS/MS transition chromatograms of the analyzed analytes are shown in Figure 1.

Fourier transform infrared (FTIR) spectroscopy was employed to characterize the NADES and verify hydrogen-bond interactions between its components, using a Nicolet 50 instrument (Thermo Scientific, USA) in attenuated total reflection (ATR) mode.

### 2.3. Preparation of Natural Deep Eutectic Solvents (NDESs)

To identify the most suitable NADES systems for extraction, various binary NADES combinations were evaluated, including anisaldehyde with acetic acid, formic acid, octanoic acid, oleic acid, thymol, and menthol. The components were mixed in a 1:1 molar ratio and stirred continuously at 70 °C until a clear, homogeneous solution was formed. The prepared NADESs were then cooled to room temperature and stored in a desiccator until further use.

### 2.4. Procedures of the Optimized NADES-DLLME Method

An aliquot of 5 mL of the sample solution was transferred into a 15 mL polypropylene centrifuge tube. Sodium chloride (10% *w*/*v*) was added and the mixture was vortexed until fully dissolved. Then, 150 µL of the selected NADES (anisaldehyde and octanoic acid) was introduced and vortexed for 1 min, producing a cloudy solution. The mixture was centrifuged at 3500 rpm for 10 min, after which the aqueous phase was carefully removed and discarded. The remaining organic layer was diluted with 100 µL of ethanol and thoroughly mixed. The solution was subsequently filtered through 0.2 µm nylon nano filter vials (Restek^®^, Bellefonte, PA, USA), and 2 µL of the prepared solution was injected into the LC-MS/MS system for analysis.

### 2.5. Method Validation

The developed NADES-DLLME-UHPLC/MS-MS method was validated by evaluating linearity, limits of detection (LODs), limits of quantitation (LOQs), accuracy and precision. To matrix effects assessment, blank samples of each matrix (milk and eggs) that are free of the target analytes were used. Recovery and precision were determined by spiking these blanks with the target analytes at three concentration levels (n = 6).

For every analytical batch, a procedural blank prepared with ultrapure water containing no added analytes was included in the analysis after every ten samples to ensure the absence of contamination. Matrix-matched calibration curves were prepared by spiking homogenized blank samples of each matrix with eight different concentrations of the target antibiotics. The spiked samples were extracted following the previously described procedure. Linearity was evaluated by calculating regression parameters (slope, intercept, and regression coefficient) for each analyte in the milk and egg matrices. LODs and LOQs were estimated based on signal-to-noise ratios of 3:1 and 10:1, respectively.

Method accuracy was assessed by triplicate analysis of spiked blank samples at three concentration levels. Recovery (%) for each analyte in each matrix was calculated as the ratio of the recovered amount to the known spiked amount. Method accuracy was assessed by triplicate analysis of spiked blank samples at three concentration levels. Recovery (%) for each analyte in each matrix was calculated as the ratio of the recovered amount to the known spiked amount. Intra-day precision and Inter-day precision were evaluated by analyzing six samples (using the area ratios of analytes to internal standards) at three concentration levels on the same day and across three separate days, respectively, and %RSD was calculated for each analyte in each matrix.

### 2.6. Sample Collection and Preparation

The method was further applied to analyze the target analytes in food products obtained from the Saudi market. A total of 44 samples, comprising 24 chicken eggs and 20 bottled cow milk samples of local and international brands were purchased from supermarkets in Dammam, Saudi Arabia between April and May 2024. All samples were promptly transported to the laboratory in ice boxes to preserve their integrity and were assigned unique codes.

For sample preparation, 2.0 g of homogenized whole egg or 10 mL of milk was transferred into a 50 mL polypropylene centrifuge tube. Each sample was spiked with 250 µL of a 1000 µg L^−1^ internal standard solutions and vortex-mixed thoroughly.

For egg samples, 2 mL of ethanol and 250 µL of 0.6% acetic acid were added, followed by 1 min of sonication. For milk samples, 2 mL of 0.6% acetic acid and 4 mL of ethanol were sequentially added, with 1 min of shaking after each addition. All samples were centrifuged at 3500 rpm for 10 min, and the resulting supernatants were quantitatively transferred into 25 mL volumetric flasks. The volumes were adjusted to the mark with 0.2 M ammonium acetate buffer (pH 5), followed by vortex mixing. The prepared solutions were then subjected to DLLME.

## 3. Results and Discussion

### 3.1. FTIR Spectra

**Figure 2:**
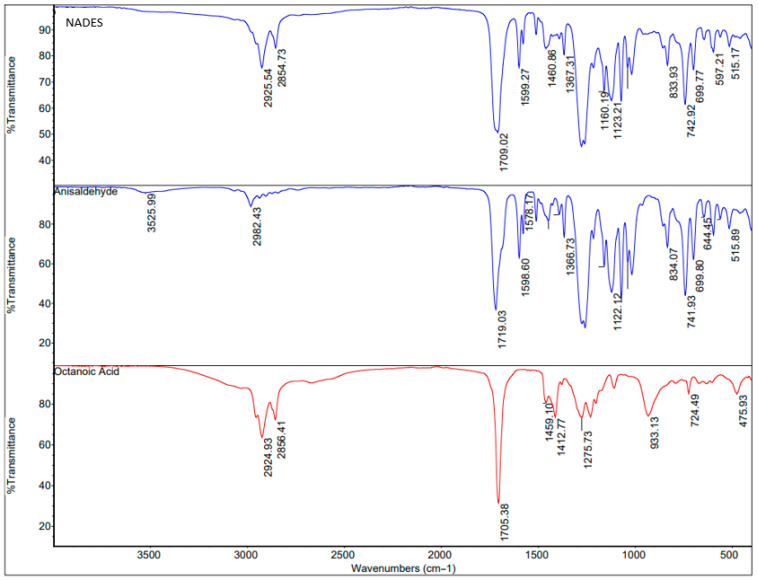
FTIR spectra of anisaldehyde, octanoic acid and the synthesized anisaldehyde-octanoic acid NADES.

To elucidate the structural features of the prepared NADES and the hydrogen-bonding interactions among its constituents, FTIR spectra of anisaldehyde, octanoic acid, and their resulting NADES were recorded, as presented in Figure 2. In the FTIR spectrum of anisaldehyde, a distinct absorption band associated with the asymmetric stretching of aromatic C–H appeared at 2982.43 cm^−1^. Characteristic stretching vibrations of the C=O, C=C, and C–C bonds were observed at 1719.03, 1598.60, and 1578.17 cm^−1^, respectively. Likewise, the FTIR spectrum of octanoic acid (Figure 2) exhibited a broad band within the 2600–2500 cm^−1^ range, corresponding to the carboxylic –OH stretching vibration. Additional absorption peaks were found at 2924.93 cm^−1^ (CH_2_ symmetric stretching), 2856.41 cm^−1^ (CH_2_ asymmetric stretching), and 1705.38 cm^−1^ (C=O stretching). Several peaks within 1200–1400 cm^−1^ were attributed to CH wagging vibrations, while the band at 933.13 cm^−1^ was assigned to the out-of-plane bending of dimeric C–O–H···O=C intermolecular hydrogen bonds. The FTIR spectrum of the NADES derived from anisaldehyde and octanoic acid (Figure 2) displayed notable spectral alterations. The broad –OH stretching band (2600–2500 cm^−1^) and the dimeric C–O–H···O=C bending signal at 933.13 cm^−1^ disappeared, indicating a disruption of the acid dimer structure. Furthermore, the C=O stretching band of octanoic acid shifted slightly to approximately 1709.02 cm^−1^. These spectral variations confirm the breakdown of octanoic acid dimerization and suggest the formation of new hydrogen-bonding interactions between the carboxylic –OH group and anisaldehyde.

### 3.2. Optimization of the Developed NADES-DLLME Method

To achieve high extraction efficiency, the NADES-DLLME method was systematically optimized. A one-variable-at-a-time approach was employed to optimize key extraction parameters because it allows for a clear understanding of the individual effect of each parameter, is simple to implement without specialized software, and reduces experimental complexity. It also facilitates direct observation of trends and potential interactions, which can guide further refinements. The parameters used for optimization included the NADES type, molar ratio, volume, sample volume, salt concentration, pH, vortex time, and centrifugation time

#### 3.2.1. Selection of NADES Type, Molar Ratio and Volume

**Figure 3:**
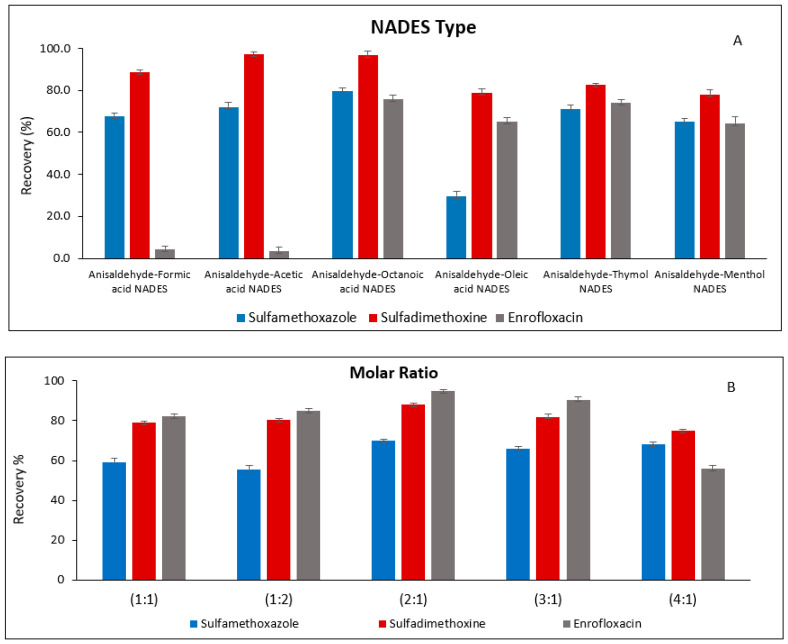
Effect of (**A**) NADES type and (**B**) Molar ratio of anisaldehyde-octanoic acid NADES on the extraction recovery of the studied analytes.

The choice of a suitable NADES is critical for maximizing analyte enrichment in DLLME. In this study, six NADES systems were prepared, combining anisaldehyde as a common component with various acids (acetic acid, formic acid, octanoic acid, oleic acid) and two monoterpenes (thymol and menthol). Initially, all NADESs were prepared at a 1:1 molar ratio following the previously described procedure. All systems produced clear, homogeneous liquids at room temperature without crystallization, indicating successful NADES formation. The extraction efficiency of each NADES was evaluated for the target analytes. Among the tested systems, anisaldehyde–octanoic acid exhibited the highest extraction recovery (Figure 3A) and was therefore selected as the extraction solvent for subsequent experiments. To determine the optimal molar ratio, anisaldehyde–octanoic acid NADES was tested at various ratios (1:1, 1:2, 2:1, 3:1, and 4:1). The 2:1 ratio provided the best extraction performance (Figure 3B) and was adopted for all DLLME procedures.

**Figure 4:**
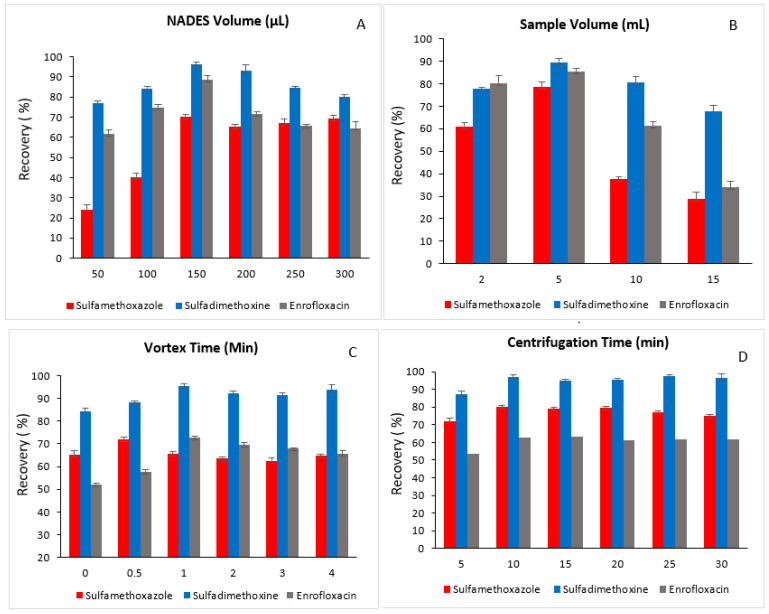
Effect of (**A**) NADES volume, (**B**) sample volume, (**C**) vortex time and (**D**) centrifugation time on the extraction recovery of the studied analytes.

To determine the optimal NADES volume, several volumes ranging from 50 to 300 µL were tested. As shown in Figure 4A, the extraction efficiency increased when the NADES volume was raised from 50 µL to 150 µL, but further increases in volume led to a decline in recovery. This decrease at higher volumes is likely due to a dilution effect, which reduces the effective analyte concentration in the NADES phase. Accordingly, a NADES volume of 150 µL was selected for all subsequent extraction procedures.

#### 3.2.2. Selection of Sample Volume

The sample volume plays a key role in the developed method’s extraction efficiency. To determine the optimal volume, sample amounts ranging from 2 to 15 mL were evaluated. The highest recoveries for the studied analytes were achieved with a 5 mL sample (Figure 4B). Volumes greater than 5 mL resulted in decreased recoveries, likely due to a dilution effect. Therefore, a sample volume of 5 mL was selected for the extraction of real samples.

#### 3.2.3. Selection of Vortex Time and Centrifugation Time

The vortexing step is essential for enhancing the mass transfer of analytes into the NADES phase. To determine the optimal time, vortex durations of 0, 0.5, 1, 2, 3, and 4 min were tested. As shown in Figure 4C, extraction recovery increased when the vortex time was extended from 0 to 1 min. Further increases beyond 1 min did not significantly improve recovery, indicating that the extraction equilibrium had already been achieved. Therefore, 1 min was selected as the optimal vortex time.

Centrifugation time was evaluated over a range of 5–30 min at a constant speed of 3500 rpm. Recovery improved when centrifugation was increased from 5 to 10 min, while longer durations did not produce further enhancement (Figure 4D). Accordingly, a centrifugation time of 10 min was chosen for all subsequent extractions.

#### 3.2.4. Selection of pH

**Figure 5:**
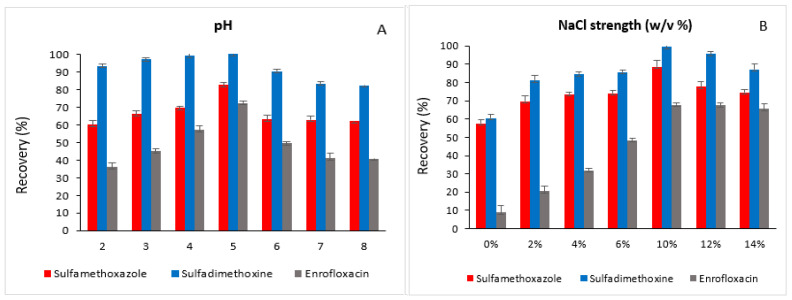
Effect of (**A**) pH and (**B**) NaCl strength on the extraction recovery of the studied analytes.

The effect of sample pH on the extraction of sulfamethoxazole, sulfadimethoxine, and enrofloxacin was evaluated over the range of 2–8. As shown in Figure 5A, the extraction efficiency reached its maximum at pH 5, while higher pH values led to a noticeable decline in recoveries. The pH 5 was therefore selected as the optimal condition for all subsequent extractions. This can be explained by the ionization behavior of the analytes: sulfamethoxazole (pKa~5.7) and sulfadimethoxine (pKa~6.9) are predominantly neutral, and enrofloxacin (pKa_1_~6.2, pKa_2_~8.5) is minimally ionized at this pH. Maintaining slightly acidic conditions increases the analytes’ affinity for the hydrophobic NADES phase, enhancing partitioning and extraction efficiency.

#### 3.2.5. Selection of Salt Concentration

The influence of salt addition on the extraction efficiency of the developed NADES-DLLME method was evaluated by adding different amounts of NaCl (0–14% *w*/*v*) to the sample solution while keeping all other conditions constant. As illustrated in Figure 5B, the extraction efficiency increased with increasing salt concentration up to 10%, after which further increases in NaCl did not significantly improve the recoveries. This enhancement is attributed to the salting-out effect, which reduces the solubility of the analytes in the aqueous phase and promotes their partitioning into the NADES phase. Accordingly, a salt concentration of 10% (*w*/*v*) was selected as the optimal condition for subsequent extractions.

### 3.3. Method Validation

The analytical performance of the developed NADES-DLLME-UHPLC/MS-MS method was thoroughly validated in terms of linearity, sensitivity, precision, and accuracy following international guidelines.

Matrix-matched calibration curves were built for cow milk (0.1–50 µg L^−1^) and chicken eggs (0.6–125 µg kg^−1^) samples. Excellent linearity was achieved, with determination coefficients (r^2^) of ≥0.9962 for cow milk and ≥0.9995 for chicken egg (Table 2), demonstrating the method’s suitability for quantitative analysis over the tested concentration ranges. The limits of detection (LODs) and limits of quantification (LOQs) were determined based on signal-to-noise ratios of 3:1 and 10:1, respectively. LODs for the investigated analytes ranged from 0.01–0.07 µg L^−1^ in cow milk and 0.06–0.38 ng Kg^−1^ in chicken egg, highlighting the method’s high sensitivity for trace-level detection in complex food matrices. Intra-day (n = 6) and inter-day (n = 6) precision were evaluated at three concentration levels. The relative standard deviations (RSDs) were ≤6.04% for cow milk and ≤5.59% for chicken egg samples, respectively, indicating excellent repeatability and reproducibility of the extraction and quantification procedures. These results also confirm the stability of the prepared NADES during use and the reproducibility of its extraction efficiency between independently prepared batches. Method accuracy was assessed through recovery experiments at three spiking levels, with absolute extraction recoveries ranging from 80.7% to 93.9%, confirming the strong extraction efficiency and quantitative reliability of the NADES-DLLME method for sulfamethoxazole, sulfadimethoxine, and enrofloxacin in both cow milk and chicken egg matrices. Overall, the developed method exhibits robust analytical performance, combining high sensitivity, precision, and accuracy, making it suitable for routine monitoring of antibiotic residues in food products. The main analytical characteristics are summarized in Table 2.

### 3.4. Analysis of Real Samples

To assess the influence of chicken egg and cow milk matrices on the analytical performance of the developed method, real samples were spiked with the target analytes at three concentration levels. Each fortified sample was analyzed in triplicate, and the corresponding percentage recoveries were calculated. The recoveries ranged from 89.1–97.9% with relative standard deviations (%RSD) ≤ 5.8% for chicken egg samples, and from 89.8–99.1% with %RSD ≤ 5.4% for cow milk samples, confirming the method’s satisfactory accuracy and precision (Table 3).

**Figure 6:**
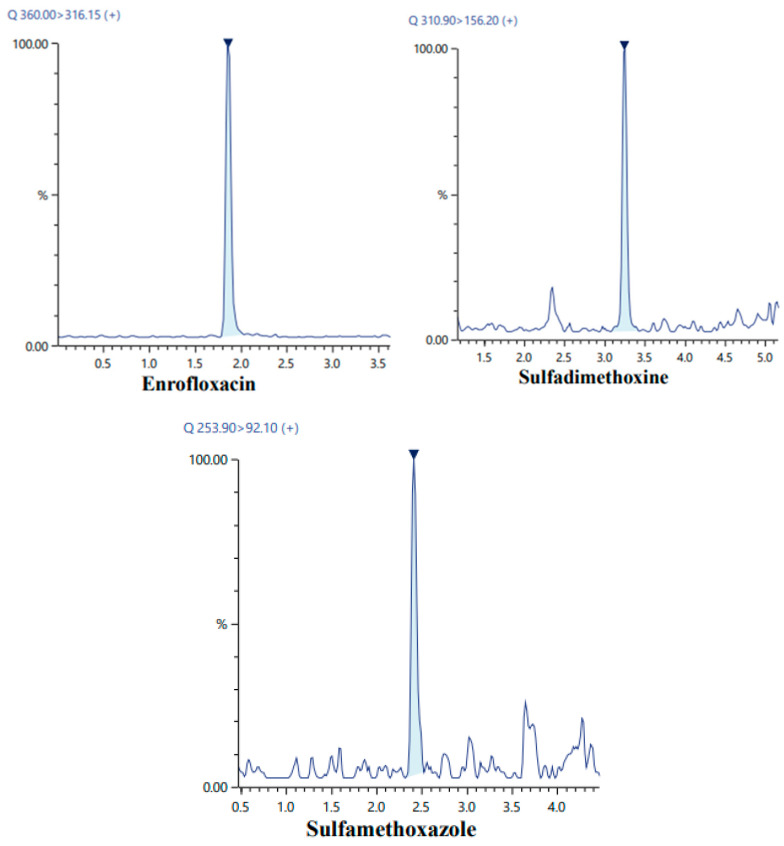
A representative LC–MS/MS transitions of the studied analytes in a milk sample (No. 29).

The developed NADES-DLLME-UHPLC/MS-MS method was successfully applied to determine sulfamethoxazole, sulfadimethoxine, and enrofloxacin residues in 20 chicken egg and 24 cow milk samples from the Saudi market. Representative LC–MS/MS transitions of the studied analytes in a real sample using the developed method is presented in Figure 6.

In chicken egg samples, sulfamethoxazole was the most frequently detected antibiotic, appearing in 90% of the samples, with concentrations ranging from 2.17 to 13.76 µg kg^−1^ (mean: 7.20 µg kg^−1^). Sulfadimethoxine was present in 80% of egg samples, at 0.14–12.65 µg kg^−1^ (mean: 5.23 µg kg^−1^), while enrofloxacin was detected in 45% of the samples, ranging from 0.67 to 13.46 µg kg^−1^ (mean: 6.64 µg kg^−1^). All observed concentrations were well below the established MRLs (100 µg kg^−1^ for all three antibiotics), indicating that the analyzed egg samples are safe for consumption regarding these residues.

In cow milk samples, sulfamethoxazole was detected in 70.8% of samples, at levels between 0.26 and 26.67 µg L^−1^ (mean: 5.94 µg L^−1^). Sulfadimethoxine appeared in 20.8% of samples, ranging from 0.53 to 2.29 µg L^−1^ (mean: 1.35 µg L^−1^), while enrofloxacin was found in 41.7% of samples, with concentrations of 0.90–171.82 µg L^−1^ (mean: 22.59 µg L^−1^). Except for a single enrofloxacin sample exceeding the MRL of 100 µg L^−1^. However, all detected residues were within permissible limits, the occasional exceedance emphasizes the need for careful veterinary use and continuous monitoring to ensure consumer safety. The results are presented in Table 4.

Overall, sulfamethoxazole exhibited the highest detection frequency and consistent presence in both egg and milk samples, reflecting its common use in animal husbandry. Sulfadimethoxine was less prevalent, whereas enrofloxacin, although moderately frequent, presented the highest concentration detected in milk, indicating potential misuse in dairy production. These findings indicate that, in general, egg and milk samples analyzed in this study are compliant with international standards and pose minimal risk to consumers. However, the elevated enrofloxacin concentration in one cow milk sample suggests that strict monitoring and adherence to withdrawal periods are essential to avoid exceeding regulatory limits.

In future studies, the developed DLLME-UHPLC method will be expanded to include additional antibiotic analytes, and the applicability of the anisaldehyde-based NADES will be explored in diverse food matrices, including meat and chicken, to further demonstrate its versatility and potential for broader food safety monitoring.

### 3.5. A Comprehensive Assessment of the Method’s Greenness, Applicability, Performance and Innovation

The developed method was comprehensively evaluated in terms of its environmental sustainability, practical applicability, analytical performance, and degree of innovation. To ensure a multidimensional assessment, ten complementary metrics were applied. The integration of these diverse evaluation frameworks provided a holistic appraisal of the method’s profile and confirmed its alignment with the modern principles of green and sustainable analytical science.

#### 3.5.1. Assessment of the Environmental Impact Associated with the Proposed Method and Compliance with the Principles of GAC

To advance green pharmaceutical analysis, several strategies have been proposed to achieve this objective, including reduction in harmful solvents consumption and waste production, employing environmentally benign solvents and reagents, and miniaturizing sample preparation and separation processes without compromising chromatographic efficiency [33,34]. In this study, the developed method incorporated GAC principles in its design, employing a short sub-2 µm narrow-bore column for rapid and efficient chromatographic separation, and using ethanol and water as mobile-phase solvents due to their low environmental impact. Sample preparation relied on micro-scale extraction with a natural, benign solvent (anisaldehyde), further minimizing reagent consumption and waste generation. To comprehensively evaluate the greenness profile of the developed NADES-DLLME-UHPLC–MS/MS method, six greenness assessment tools were applied to assess its environmental impact, safety, and potential implications for human health. These included the Analytical Eco-Scale (AES), the Green Analytical Procedure Index (GAPI), the Analytical GREEnness Metric (AGREE), the Analytical GREEnness Metric for Sample Preparation (AGREEprep), the Modified Green Analytical Procedure Index (MoGAPI), and the Analytical Green Star Area (AGSA) framework. Together, these assessments can provide a multidimensional perspective on the method’s compliance with the principles of GAC.

**Figure 7:**
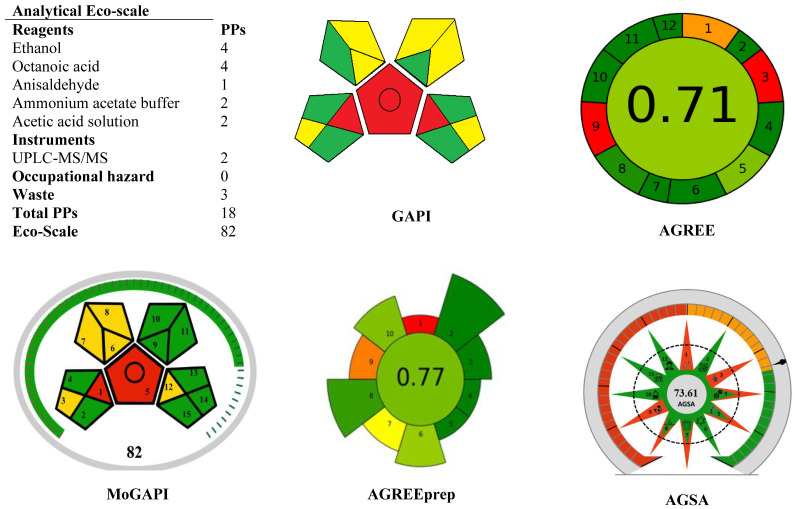
The eco-friendliness profile of the developed method using Eco-scale, GAPI, AGREE, MoGAPI, AGREEprep and AGSA tools.

The AES tool assigns penalty points for various parameters such as the type and volume of reagents used, occupational hazards, waste generation, and energy consumption. The total score is calculated by subtracting penalty points from a maximum of 100. Based on this framework, methods with scores > 75 are classified as excellent green methods, those between 50–75 as acceptable green, and those <50 as inadequately green [23]. In this study, the Analytical Eco-Scale score of 82 classifies the method as highly green, reflecting the use of a natural deep eutectic solvent (anisaldehyde–octanoic acid), minimal reagent consumption and reduced waste generation (Figure 7).

GAPI offers a more holistic visualization of the greenness of the method across its entire analytical workflow. It is represented in a five-field pictogram, where each field corresponds to a specific procedural step. The fields are color-coded as green (low ecological impact), yellow (medium impact), or red (high impact), with a higher prevalence of green zones reflecting a lower environmental burden. Further details about GAPI design and interpretation are available in reference [24]. For the presented method, GAPI analysis highlighted six green zones, indicating low environmental impact across critical steps such as sample preparation, solvent use, and waste handling, while only three red zones were observed, confirming the overall eco-friendliness of the method (Figure 7).

AGREE tool complements these evaluations by providing a circular pictogram divided into twelve sections, each corresponding to one of the twelve principles of green analytical chemistry. Each section is color-scaled from red to green, reflecting the degree of compliance, while the central zone displays the overall greenness score on a scale from 0 (non-green) to 1 (fully green). The tool thus provides both qualitative and quantitative insights into the eco-friendliness of the method [25]. The AGREE metric yielded a score of 0.65 (Figure 7), demonstrating good alignment with the 12 principles of GAC, particularly due to the use of renewable solvents, low sample and solvent volumes.

AGREEprep is a metric specifically designed to evaluate the environmental sustainability of sample preparation procedures in analytical chemistry [26]. It expands upon the principles of the original AGREE tool by focusing on the pre-analytical stage, where the majority of waste generation, solvent consumption, and energy use typically occur. AGREEprep assesses the greenness of a sample preparation method through a set of well-defined criteria based on the 10 principles of green sample preparation. These criteria consider factors such as sample amount, solvent type and volume, energy efficiency, automation, waste management, and operator safety. The software provides a visual, color-coded score (ranging from 0 to 1) that reflects the overall sustainability of the sample preparation process. In this study, AGREEprep score of 0.77 (Figure 7) indicates a high level of method greenness [26].

To strengthen the evaluation, MoGAPI was also applied. Unlike the classical GAPI, MoGAPI incorporates a more detailed scoring system that quantifies the degree of greenness and integrates a numerical value with the visual representation. This modification allows for enhanced differentiation between methods with similar GAPI patterns, making it especially useful for comparative assessment [27]. The MoGAPI assessment produced a score of 82 (Figure 7) which emphasizes low chemical hazards and minimal environmental footprint and consistent with the Eco-Scale results and confirming the method’s environmentally responsible design.

In addition, the recently introduced AGSA framework was used. AGSA builds on the AGREE approach by converting the greenness pictogram into a quantitative area-based score, which enables more accurate comparisons between analytical methods. This geometric representation highlights not only compliance with green chemistry principles but also the extent of sustainability improvements achieved [28]. The method’s AGSA score is 73.61 (Figure 7) which corroborated the findings of other metrices and broad green coverage across solvent safety, energy efficiency, and waste reduction.

#### 3.5.2. Evaluation of the Method’s Applicability and Practicality

For assessing the applicability and practicality of the developed method, the Blue Assessment of Green Index (BAGI) [29] and the Click Analytical Chemistry Index (CACI) [30] were used.

BAGI is a recently developed tool that extends the evaluation of analytical methods beyond environmental criteria by integrating additional dimensions of sustainability, namely economic and social factors [29]. This comprehensive index provides an overall measure of a method’s alignment with sustainability principles. The developed method achieved a BAGI score of 70, confirming its strong conformity with the broader concept of sustainable analytical chemistry (Table 5), with benefits extending to both environmental protection and analytical practice.

CACI is a user-friendly framework designed to assess the feasibility and practicality of analytical methods. It offers a digital platform for comparing methodologies based on their sustainability, applicability, and effectiveness. By emphasizing innovation and future adaptability, the CACI approach ensures that emerging methods meet both scientific and industrial expectations [30]. The proposed method obtained a CACI score of 80, indicating satisfactory performance and high practical suitability (Table 5).

#### 3.5.3. Evaluation of the Method’s Analytical Performance

To evaluate the analytical performance of the developed method, the Red Analytical Performance Index (RAPI) [31] was applied. RAPI is a comprehensive metric that assesses the overall performance and reliability of analytical procedures [31]. It systematically examines key validation parameters in accordance with the principles of white analytical chemistry. The proposed method achieved a RAPI score of 82.5, confirming its excellent analytical quality and robustness (Table 5).

#### 3.5.4. Evaluation of the Method’s Analytical Innovation

For evaluating the method’s level of innovation, the Violet Innovation Grade Index (VIGI) [32] was applied. VIGI is a recently introduced evaluation tool designed to quantify the degree of innovation in analytical methodologies. It incorporates ten key criteria, encompassing aspects such as sample preparation, instrumentation, data processing, chemical usage, and alignment with the principles of WAC. This comprehensive framework complements and harmonizes the existing green, blue, and red sustainability metrics [32]. In this study, the developed method achieved a VIGI threshold score of 75 (Table 5), indicating its classification as an innovative analytical approach.

In conclusion, the developed method demonstrates excellent environmental performance, particularly in the sample preparation stage, where the use of natural deep eutectic solvents (DESs) and microextraction greatly enhances its sustainability. A comprehensive evaluation using ten assessment tools confirmed that the NADES-DLLME approach is both analytically robust and environmentally responsible. It offers a safe, economical, and sustainable alternative to conventional extraction techniques, fully aligned with the principles of modern green and white analytical chemistry and ideally suited for food safety monitoring.

### 3.6. Comparison to Other Reported Methods

Various methods have been reported for the determination of antibiotic residues in milk and egg samples, utilizing different sample extraction techniques [35,36,37,38,39]. In comparison with these methods, the developed anisaldehyde–octanoic acid NADES-DLLME method coupled with UHPLC–MS/MS for the determination of sulfamethoxazole, sulfadimethoxine, and enrofloxacin in cow milk and chicken egg samples demonstrated excellent analytical performance. Recoveries, intra- and inter-day precision, and determination coefficients were comparable to or better than those reported for conventional methods such as QuEChERS, liquid–liquid extraction, and DLLME. The combination of NADES-DLLME with UHPLC–MS/MS provides high sensitivity and superior chromatographic performance compared to LC–UV approaches (see Table 6).

A major advantage of the proposed method is its greenness: unlike previously reported methods that rely on hazardous, toxic, or non-biodegradable solvents, the NADES-DLLME approach employs benign alternatives such as water, ethanol, and anisaldehyde, thereby reducing environmental impact. The extraction is rapid, requires minimal solvent, and avoids complex cleanup steps, resulting in short run times and high throughput while maintaining robust quantification in complex milk and egg matrices. In conclusion, compared with documented approaches, the proposed method is faster, more eco-friendly, cost-effective, and operationally simpler, without compromising sensitivity, accuracy, or precision. The integration of NADES-DLLME with UHPLC–MS/MS offers a sustainable, affordable, and high-performance alternative for routine monitoring of the studied antibiotics in food of animal origin.

## 4. Conclusions

In this study, a rapid, sensitive, and environmentally sustainable method based on NADES-DLLME coupled with UHPLC–MS/MS was successfully developed and validated for the simultaneous determination of sulfamethoxazole, sulfadimethoxine and enrofloxacin in chicken egg and cow milk samples from the Saudi market. The method exhibited excellent analytical performance, including high linearity (r^2^ ≥ 0.9962), low limits of detection (0.06–0.38 µg kg^−1^ for eggs, 0.01–0.07 µg L^−1^ for milk), satisfactory recoveries (89.5–98.7%), and excellent intra- and inter-day precision (RSDs ≤ 6.04%).

Application of the method to real samples demonstrated the presence of the target antibiotics in several egg and milk samples, with mean concentrations remaining below the established maximum residue limits (MRLs) for food safety, highlighting its relevance for routine monitoring and regulatory compliance. Sulfamethoxazole was the most frequently detected antibiotic, followed by enrofloxacin and sulfadimethoxine, reflecting the patterns of veterinary antibiotic use. Importantly, the developed method aligns with the principles of GAC. The use of a NADES composed of anisaldehyde and octanoic acid eliminated the consumption of hazardous organic solvents and reduced waste generation. A comprehensive assessment using ten metrics confirmed the method’s eco-friendliness, sustainability, practicality, and innovation, reinforcing its suitability for routine food safety analysis supporting both public health protection and sustainable analytical practices.

## Figures and Tables

**Table 1:** Retention times and MS/MS parameters for the studied analytes.

Analyte	Sulfamethoxazole	Sulfadimethoxine	Enrofloxacin	Sulfamethoxazole (Phenyl-^13^C_6_)	Ofloxacin-d3
Retention Time (min)	2.51	3.28	1.85	2.51	1.68
Precursor ion *m*/*z*	253.9	310.9	360	260	360
Product ion 1 *m*/*z*	92.1	156.2	316.2	114.3	316.2
Collision energy (eV)	−27	−21	−19	−26	−19
Pause Time (msec)	3	3	3	3	3
Dwell Time (msec)	44	44	44	44	44
Product ion 2 *m*/*z*	156.1	92.1	342.2	162.2	342.2
Collision energy (eV)	−15	−32	−22	−16	−22
Q1 Pre Bias (V)	12	12	10	13	10
Q3 Pre Bias (V)	16	17	23	11	23

**Table 2:** Analytical performance of the developed NADES-DLLME-UHPLC method.

Sample	Chicken Egg (n = 20)			Cow Milk (n = 24)		
Analytes	Sulfamethoxazole	Sulfadimethoxine	Enrofloxacin	Sulfamethoxazole	Sulfadimethoxine	Enrofloxacin
Linearity range	0.6–125	0.6–125	0.6–125	0.1–50	0.1–50	0.1–50
Regression Equation						
Slope	5.34 × 10^−2^	17.6 × 10^−2^	5.57 × 10^−2^	5.06 × 10^−1^	18.3 × 10^−1^	7.38 × 10^−1^
Intercept	8.16 × 10^−3^	2.56 × 10^−2^	3.32 × 10^−2^	6.19 × 10^−3^	2.19 × 10^−2^	1.19 × 10^−2^
Determination Coefficient (r^2^)	0.9995	0.9997	0.9998	0.9983	0.9996	0.9982
LOD	0.13	0.07	0.06	0.02	0.01	0.01
LOQ	0.38	0.21	0.18	0.07	0.03	0.03
Intra-day precision	R% ± RSD			R% ± RSD		
High level	89.5 ± 4.80	89.8 ± 2.41	92.7± 5.08	90.9 ± 2.81	89.9 ± 3.69	93.9 ± 2.70
Medium level	98.5 ± 5.32	96.8 ± 2.61	90.7± 3.22	98.7 ± 5.65	95.6 ± 4.76	91.5 ± 2.24
Low level	90.7 ± 5.02	93.9 ± 4.70	94.8± 3.59	91.8 ± 2.79	98.4 ± 6.04	89.7 ± 4.06
Inter-day precision	R% ± RSD			R% ± RSD		
High level	92.9 ± 4.72	90.9 ± 3.71	93.5 ± 4.12	89.5 ± 3.09	90.34 ± 4.26	91.51 ± 2.55
Medium level	90.7 ± 5.59	97.3 ± 2.59	89.8 ± 4.27	92.24 ± 5.35	94.27 ± 6.01	90.9 ± 2.02
Low level	89.8 ± 5.07	91.4 ± 4.29	90.9 ± 2.97	95.32 ± 4.77	93.62 ±4.89	89.9 ± 3.75

Concentrations are expressed as µg kg^−1^ for chicken egg and µg L^−1^ for cow milk samples. High, medium, and low spiking levels were 1, 50, and 100 µg kg^−1^ for chicken egg, and 0.5, 10, and 40 µg L^−1^ for cow milk samples, respectively.

**Table 3:** Results of the analysis of chicken egg and cow milk samples spiked with the investigated analytes (n = 3).

Analyte	Spiked Level (µg Kg^−1)^	Chicken Egg		Spiked Level (µg L^−1^)	Cow Milk	
		% R	RSD (%)		% R	RSD (%)
Sulfamethoxazole	100	97.9	2.1	40	94.9	4.9
	5	91.5	3.4	10	90.2	5.4
	1	90.5	5.3	0.5	97.3	4.7
Sulfadimethoxine	100	95.9	4.6	40	89.8	3.2
	5	90.1	5.2	10	91.5	2.8
	1	89.1	3.9	0.5	98.4	4.1
Enrofloxacin	100	92.5	4.4	40	90.8	3.5
	5	91.9	5.8	10	92.7	2.4
	1	93.2	3.2	0.5	99.1	4.9

**Table 4:** Results of the analysis of chicken egg and cow milk samples (n = 44).

Sample No.	Sample Type	Sulfamethoxazole	Sulfadimethoxine	Enrofloxacin
1	Chicken egg * (n = 20)	6.12	0.32	nd
2		5.35	0.14	nd
3		5.73	nd	nd
4		nd	nd	nd
5		6.97	10.53	nd
6		11.16	12.65	nd
7		9.07	11.59	nd
8		5.74	4.42	13.46
9		8.82	5.70	11.49
10		5.74	5.74	12.48
11		13.76	11.48	nd
12		10.40	5.93	nd
13		12.08	8.71	nd
14		2.17	0.90	1.40
15		3.25	2.17	5.48
16		nd	nd	nd
17		5.44	2.22	3.29
18		3.27	0.85	10.25
19		10.19	0.27	0.67
20		4.29	nd	1.28
21	Cow milk ** (n = 24)	nd	nd	nd
22		nd	nd	nd
23		nd	nd	nd
24		nd	nd	nd
25		nd	nd	nd
26		4.04	2.29	11.41
27		3.51	1.48	17.99
28		3.77	1.89	14.70
29		0.37	0.57	4.65
30		nd	nd	nd
31		nd	nd	nd
32		1.25	nd	nd
33		1.62	nd	0.90
34		1.43	nd	0.92
35		0.26	0.53	nd
36		1.87	nd	1.26
37		1.38	nd	1.02
38		1.62	nd	1.26
39		1.60	nd	171.82
40		14.41	nd	nd
41		22.80	nd	nd
42		26.67	nd	nd
43		7.48	nd	nd
44		6.90	nd	nd

* µg Kg^−1^, ** µg L^−1^, and nd: not detected.

**Table 5:** Evaluation of the developed method’s applicability, performance and innovation.

**Applicability**	
**BAGI**	**CACI**
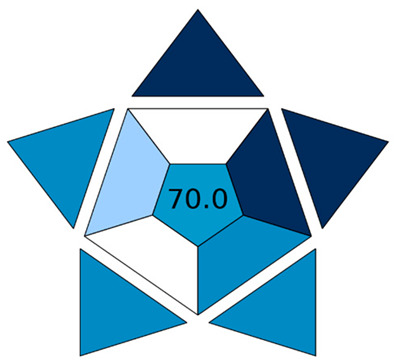	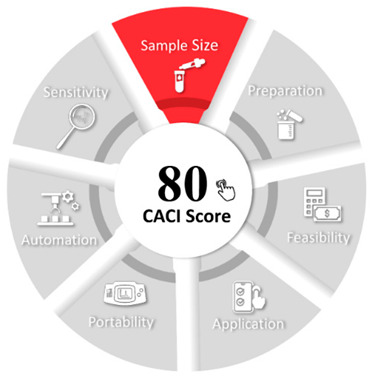
**Performance**	**Innovation**
**RAPI**	**VIGI**
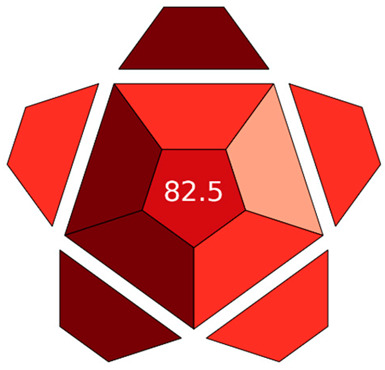	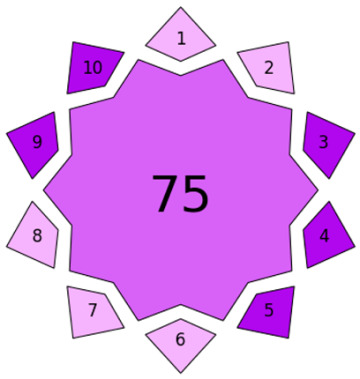

**Table 6:** Comparison of the proposed NADES-DLLME-UPLC/MS-MS method with other reported methods for determination of antibiotics in milk and egg samples.

Matrix	Separation Technique	Extraction Method	Solvents and Reagents	Linearity Range	R^2^	LOD	Recovery	RSD	Refs.
Egg	UPLC-MS/MS	Solvent extraction	Acetonitrile and formic acid	10–2500 µg kg^−1^	≥0.990	2–10 µg kg^−1^	80.0–128.01%	≤13.97%	[35]
Egg and chicken	HPLC-UV	water:ethyl acetate (1:3, *v*/*v*) liquid-liquid extraction.	Acetic acid, methanol and acetonitrile	50–250 µg Kg^−1^	˃0.99	129–140 µg Kg^−1^	86–108%	˂15%	[36]
Milk	HPLC-UV	QuEChERS	Acetic acid and acetonitrile	12.5–200 µg L^−1^	≥0.995	0.2 µg L^−1^	>90%	≤12.79	[37]
Milk	UPLC-UV	Stir bar sorptive extraction	1-dodecanol, methanol and acetonitrile	10–1000 µg L^−1^	˃0.996	1.30–7.90 µg L^−1^	54.8–126%	≤10.9	[38]
Milk	HPLC-Fluorescence	DLLME	Methanol, ethanol, acetonitrile, sodium chloride, dichloromethane, sodium hydroxide, acetic acid, chloroform and hydrochloric acid	2.01–250 µg L^−1^ 3.85–250 µg L^−1^	≥0.993 ≥0.992	˂1.21 µg L^−1^ ˂2.73 µg L^−1^	83.6–104.8%	≤9.7%	[39]
		QuEChERS					90.8–104.7%	≤9.2%	
Milk & egg	UHPLC-MS/MS	NADES-DLLME	Ethanol, anisaldehyde and octanoic acid	0.1–50 µg L^−1^ 0.6–125 µg Kg^−1^	≥0.998	0.01–0.02 µg L^−1^ 0.06–0.13 µg Kg^−1^	89.5–98.7%	≤6.04%	This study

## Data Availability

The raw data supporting the conclusions of this article will be made available by the authors on request.
